# Executive Functioning Profiles in Children with Neurodevelopmental Disorders

**DOI:** 10.3390/bs15091256

**Published:** 2025-09-14

**Authors:** Esperanza Bausela Herreras

**Affiliations:** Department of Health Sciences, Public University of Navarra, 31006 Pamplona, Spain; esperanza.bausela@unavarra.es

**Keywords:** BRIEF, dysexecutive syndrome, executive function, neurodevelopmental disorders

## Abstract

Antecedents: From a functional perspective, executive functions—such as self-regulation and meta-cognition—emerge as key dimensions affected transversally across various neurodevelopmental disorders. Aim: The aim of this study is to analyze and compare executive functioning profiles in children with various neurodevelopmental disorders, as reported by parents and teachers. It is hypothesized that children with neurodevelopmental disorders exhibit executive function deficits, as measured by the BRIEF-P, in comparison to typically developing children. Methodology: We used a non-experimental methodology and ex post facto design to perform a descriptive, cross-sectional study. Participants: The normative sample is composed of 1.979 participants with typical (normotypical) development and 205 participants belonging to a clinical sample. Measurement: The instrumental development of EFs was evaluated using BRIEF-P by key informants. Results: The highest F-values were observed in the following: (i) working memory, (a) parents [F = 195.76, *p* < 0.001] and (b) teachers: [F = 199.63, *p* < 0.001]; and (ii) Emergent Metacognition Index, (a) parents [F = 176.15, *p* < 0.001] and (b) teachers [F = 187.87, *p* < 0.001]; and (iii) Executive Function Global, (a) parents [F = 168.07, *p* < 0.001] and (b) teachers [F = 207.47, *p* < 0.001]. Conclusions: This study provides a clear framework for identifying dysexecutive syndrome. Executive functioning is one of the most important abilities, and its disruption can lead to dysexecutive syndrome.

## 1. Introduction

In “Where Do Neurodevelopmental Conditions Fit in Transdiagnostic Psychiatric Frameworks? Incorporating a New Neurodevelopmental Spectrum” Michelini et al. (2024) argue that traditional DSM-5TR and ICD-11 diagnoses—such as autism spectrum disorder, ADHD, learning disorders, and intellectual disabilities—share genetic, cognitive, and neurological foundations that justify considering them together within a “neurodevelopmental spectrum.” This dimensional and transdiagnostic perspective overcomes the limitations of categorical approaches, which often generate diagnostic overlap and fail to capture the diversity of individual profiles, some of which cannot be identified in the earliest stages of development. Michelini et al.’s (2024) proposal resonates with recent work that, from different angles, also advocates for a more integrated understanding of development. For example, the Dynamic Field Theory of Executive Function (McCraw et al., 2024) shows how the learning of verbal labels for visual features in early childhood plays a structuring role in cognitive control, aligning with the idea of shared developmental trajectories modifiable by experience. Complementarily, Pezzoli and colleagues introduce “cognitive microscopy” to more sensitively measure neurocognitive variability, and the STREAM project (Williams et al., 2024) exemplifies how to bring this knowledge into low-resource settings through scalable assessments. Taken together, these contributions suggest that understanding neurodevelopment as an interconnected continuum—rather than defined exclusively by traditional clinical diagnostic categories—opens the door to earlier, more precise, and contextually adapted interventions.

Traditionally, the approach to neurodevelopmental disorders has been shaped by a categorical logic: specific diagnoses (such as ADHD, ASD, dyslexia, etc.) that define clinical groups based on distinct diagnostic criteria (see DSM-5TR) (Hynd et al., 1991; Tannock, 2013; Wilson & Bishop, 2022). However, this approach presents significant limitations, and alternative models have been proposed (Moreno-Llanos et al., 2024). One of them is that it tends to label individuals in ways that do not always accurately reflect their functional profile or their actual intervention needs (DeYoung et al., 2024; Ousley & Cermak, 2014; Poletti et al., 2017; Snyder et al., 2023).

From a functional perspective, executive functions—such as self-regulation and metacognition—emerge as key dimensions affected transversally across various neurodevelopmental disorders (Ceruti et al., 2024; Pennington & Ozonoff, 1996). Understanding these skills beyond diagnostic labels allows for more personalized and effective interventions (Margari et al., 2016).

In the context of neurodevelopmental disorders, it is essential to define, in the Introduction, key concepts such as executive function, self-regulation, and metacognition, as they constitute central processes that are transversal to multiple diagnoses (McCraw et al., 2024; Pezzoli et al., 2023).

Dysexecutive syndrome, or executive dysfunction, is a set of impairments in executive functions, which include processes like planning, inhibiting impulses, maintaining information, and adapting to change (Jones & Graff-Radford, 2021; Rabinovici et al., 2015; Stuss & Alexander, 2000). This syndrome is not exclusive to a single disorder but appears in various neurodevelopmental conditions, such as ADHD, ASD, learning, language, and coordination disorders. Although these disorders differ, they share difficulties in executive functions, which often form the core of their symptoms (Ossenkoppele et al., 2015; Roussel et al., 2017; Tang et al., 2020).

Alterations in key executive functions are directly related to the development of self-regulation in childhood (Ben-Asher et al., 2023; Dörrenbächer-Ulrich & Bregulla, 2024). Since self-regulation is an essential component of these functions, it is crucial to understand how it manifests and develops in the early stages of the life cycle.

The development of self-regulation is closely linked to that of metacognition, involving the continuous control and adjustment of thoughts and behaviors (Bausela Herreras, 2021). Theoretical models such as those of Kopp (Kopp, 1982), Piaget (1974, cited in Barrouillet, 2015), and Vygotsky (1962, cited in Yaden & Martinez-Yaden, 2023) describe its progression from initial forms of behavioral control toward flexible, reflective internal control mediated by language.

Although there is consensus that metacognition progresses throughout schooling (Veenman et al., 2006), its development does not always follow a linear trajectory and may involve both quantitative and qualitative changes in the strategies used (Kuhn, 2000; Pappas et al., 2003). Empirical evidence shows that older children display greater monitoring, control, and internalized planning than younger children, who tend to review the execution of their plans rather than the plans themselves (Bryce et al., 2015; Whitebread & O’Sullivan, 2012).

Overall, self-regulation and metacognition, as key executive functions, constitute essential dimensions both in typical development and in various neurodevelopmental disorders (Zelazo, 2020; Wu & Was, 2023). Metacognition, like self-regulation, comprises key components that include knowledge about one’s own cognitive processes and the ability to effectively regulate them (Howard & Vasseleu, 2020; Veenman et al., 2006; Schraw et al., 2006). This conscious control of learning involves planning, monitoring, and evaluating one’s own strategies and progress, which are fundamental aspects for academic development (Diamond & Lee, 2011; Diamond, 2013; Durlak et al., 2011; Morgan et al., 2019; Zelazo, 2015, 2020; Zelazo et al., 2005). Numerous studies have shown that training in metacognitive skills and executive functions from early stages significantly improves school performance and the ability to face academic challenges (Morgan et al., 2019). Together, metacognition and self-regulation, as essential dimensions of executive functions, constitute fundamental pillars for both typical and atypical development (J. Chen et al., 2021; Graham & Harris, 2018; Karalunas & Huang-Pollock, 2011; Zelazo, 2020; Wu & Was, 2023).

To conclude this Introduction, it is important to highlight that executive function, metacognition, and self-regulation are interrelated cognitive processes that develop throughout childhood and are essential for learning (Cristofori et al., 2019), as well as for understanding deviations that occur in typical development (S.-F. F. Chen et al., 2016; Daunhauer et al., 2014; R. R. Lee et al., 2023). Although these subjects are traditionally studied separately, Roebers (2017) proposes a unifying framework of cognitive self-regulation that allows for a better understanding of their joint development by exploring their similarities and differences.

### Aim and Research Hypotheses

We rely on the study by Sadozai et al. (2024), who—based on transdiagnostic models of neurodevelopment—highlight the importance of understanding delays in executive function from a developmental perspective. This can improve the design of early and personalized interventions for all children with neurodevelopmental conditions, regardless of their specific diagnosis (Mareva et al., 2024).

The recent literature highlights the transdiagnostic nature of EF in neurodevelopmental disorders. A meta-analysis of 180 studies by (Sadozai et al., 2024) found that EF deficits are consistent across different NDDs, with a moderate effect size (g = 0.56), increasing in the presence of comorbidities and when assessments are based on informant ratings. Complementarily, D. Lee et al. (2025) reviewed 111 studies and found that attention delays are small, whereas EF delays reach a moderate effect size (g ≈ 0.64), detectable as early as the preschool stage but not in the first year of life. Likewise, the systematic review by Christoforou et al. (2023) analyzed specific EF profiles in preschool children with autism spectrum disorder (ASD) and attention-deficit/hyperactivity disorder (ADHD), highlighting similarities and differences that can inform more tailored interventions.

The aim of this study is to analyze and compare executive functioning in children with various neurodevelopmental disorders, as reported by parents and teachers.

Assessing executive functions in preschool populations through indirect measures allows us to capture the manifestation of these skills in natural contexts and from the perspective of different informants. Spataro et al. (2024) compared parent and teacher ratings on the Behavior Rating Inventory of Executive Function—Preschool Version (BRIEF-P), finding systematic discrepancies: parents tended to report greater difficulties in inhibition and emotional control, whereas teachers’ ratings were more closely associated with children’s emotional understanding and cognitive skills. These results highlight the importance of integrating multiple information sources to obtain a more complete and contextualized profile of executive functioning in early childhood.

Several studies have documented discrepancies between parent and teacher ratings of child behavior, particularly in relation to executive functions, attributable to contextual differences (home vs. school) and perceptual biases specific to each informant (Albuquerque et al., 2025; De Los Reyes & Kazdin, 2005; Tamm & Peugh, 2019; Toplak et al., 2013). In this regard, significant differences are expected between executive function scores reported by both groups (BRIEF-P), with teachers likely to report greater difficulties than parents due to higher academic demands, a less permissive stance, and classroom conditions in which group dynamics and student–teacher ratios limit individualized attention.

Executive functions—such as inhibitory control, working memory, and cognitive flexibility—are core neurocognitive processes that are impaired in various neurodevelopmental conditions (ADHD, autism spectrum disorders, and learning disorders, among others). The BRIEF-P (Gioia et al., 2016) allows for the assessment of these aspects in an early-age population, capturing difficulties that may be common to multiple disorders within the neurodevelopmental spectrum proposed by the transdiagnostic framework.

It is hypothesized that children with neurodevelopmental disorders exhibit executive function deficits, as measured by the BRIEF-P (Gioia et al., 2016), in comparison to typically developing children. Additionally, it is hypothesized that perceptions of executive functioning vary depending on the informant (parent vs. teacher).

## 2. Materials and Methods

We set out to create a non-experimental, ex post facto design, descriptive, cross-sectional development study.

### 2.1. Design

The study procedure is based on a non-experimental methodology, with an ex post facto and descriptive design in which the executive profile obtained through the BRIEF-P (Gioia et al., 2016) is analyzed in a typically developing population in comparison with a sample of children with various neurodevelopmental disorders (clinical sample).

A cross-sectional study was conducted, understood as an observational study that analyzes data from a population at a single point in time. This type of design is often used to measure the prevalence of health outcomes, understand health determinants, and describe characteristics of a population. Unlike other observational studies, it does not follow individuals over time and is useful for establishing preliminary evidence to support more advanced studies (Wang & Cheng, 2020). In this case, participants—the typically developing sample versus the clinical sample—were assessed by key informants, specifically parents and teachers.

The study compares the results obtained from children with typical development to those with various neurodevelopmental disorders (clinical sample). This approach offers a clear view of developmental differences in children’s executive functions and may enhance the design of early and personalized interventions for all children with neurodevelopmental conditions, regardless of their specific diagnosis.

The instrument used to assess executive functions was the BRIEF-P (Gioia et al., 2016), which provides information on self-regulation and other cognitive functions in children within an educational context. The sample includes children aged between 2 and 5 years, with a balanced distribution of gender and age, ensuring representation of both stages of early child development.

### 2.2. Participants

The normative sample is composed of 1.979 participants with typical (normotypical) development and 205 participants belonging to a clinical sample, all of whom took part in the adaptation and validation process of the BRIEF-P (Gioia et al., 2016).

The clinical characterization, along with executive profile analysis, is described in detail in the Spanish adaptation of (Gioia et al., 2016, pp. 106–125) and, partially, in a pilot study published on two subsamples—ASD and ADHD—(Bausela Herreras, 2020). The clinical sample considered in this work includes cases diagnosed with autism spectrum disorder (ASD), attention deficit hyperactivity disorder with or without hyperactivity (ADHD), learning disorders, communication disorders, and combined presentations involving more than one of these diagnoses.

The inclusion criteria were (normotypical) willingness to participate in the study, being between 2 and 6 years of age, and showing no signs or indications of any neurodevelopmental disorder and/or disability.

In this study, participants were assessed by different informants: 54.21% by parents and 45.78% by teachers.

It should be noted that these are not paired samples; they are independent samples. In this study, the evaluators were not matched; instead, an independent groups design was chosen due to participant availability.

Regarding how parents and teachers assess the development of executive functions, there is no agreement between them, with these differences being more evident in the older age groups. Nevertheless, the data allow us to affirm that both parents and teachers are reliable sources for identifying symptoms of executive dysfunctions (Meuwissen & Carlson, 2015; Ortiz Luna & Acle Tomasini, 2006).

Information related to other demographic data is published (Bausela Herreras & Luque Cuenca, 2017; Bausela Herreras, 2024a).

The clinical sample includes children with various neurodevelopmental disorders, primarily ADHD and ASD, since, according to the literature, these conditions are commonly associated with diverse executive function deficits, as reviewed in several studies (Zelazo, 2020).

Students were grouped according to typical versus clinical development. Table 1 shows the distribution of participants by type of sample and informants.

Table 2 shows the distribution of participants according to age groups, informant, and sample.

Table 3 shows the distribution of participants according to sex, informant, and sample.

### 2.3. Measurement

Executive function assessment with BRIEF-P is an instrument that was recently validated in Spain by (Bausela Herreras & Luque Cuenca, 2017) with the aim of evaluating its development through the observation of key informants (teachers or other habitual caregivers of the child; hetero research, self-investigation).

BRIEF-P was completed by parents, legal guardians, and teachers of children aged from 2 years to 5 years and 11 months who have had knowledge of the child for a minimum period of 6 months.

It has been used with populations exhibiting various executive dysfunctions (such as attention deficit with and without hyperactivity, autism spectrum disorder, brain injury, and Tourette syndrome, among others).

The study was carried out using individual and collective applications.

Its administration takes approximately 10–15 min.

Responses are given using a three-point Likert-type frequency scale: never, sometimes, and often.

The psychometric properties can be consulted in a previous work (Bausela Herreras & Luque Cuenca, 2017), where the relevant aspects related to reliability, validity, and other essential characteristics of the instrument used are detailed.

The BRIEF-P consists of 63 items organized into five clinical scales, three indices, a Global Executive Composite, and two validity scales (negativity and inconsistency).

The BRIEF-P provides scores on various indices (Global Index of Executive Function, Inhibitory Self-Control Index, Flexibility Index, and Emergent Metacognition Index) and scales related to EFs (inhibition, flexibility, emotional control, working memory, planning, and organization). Table 4 presents the clinical scales and indices that make up the BRIEF-P (Gioia et al., 2016).

### 2.4. Procedure

The procedure for applying the BRIEF-P (Gioia et al., 2016) by parents and teachers involves four key stages to ensure the validity and proper implementation of the test. First, collaborators must familiarize themselves with the norms, thoroughly reading them to understand the guidelines, securely storing materials, and explaining the evaluation objectives and data handling to informants. Second, participants are randomly selected from a normal sample without prior diagnoses, as are informants, including parents or teachers familiar with the child’s behavior. Third, differentiated booklets are used—one for parents and another for teachers/caregivers—and informants must complete their respective booklets while maintaining confidentiality and returning them appropriately. Finally, data are entered into the scoring program at www.teacorrige.com, generating a graphical profile, intended as a general guide rather than a clinical diagnosis.

The study was conducted between 2013 and 2016 in co-authorship with Luque and the author of the present work, as part of the Spanish validation process (Bausela Herreras & Luque Cuenca, 2017; Gioia et al., 2016).

### 2.5. Research Variables

In order to analyze the executive functioning profiles of the participants, a set of variables was defined for the study.

The independent variables include both the type of sample (normotypical development vs. clinical) and the type of informant (parents, legal guardians, and teachers). These variables are expected to influence the way executive function behaviors are reported.

The dependent variable is executive functioning, operationalized through the clinical scales and indices provided by the BRIEF-P (Gioia et al., 2016), offering a comprehensive assessment of key components such as inhibition, flexibility, emotional control, working memory, and planning/organization.

### 2.6. Analysis of Data

The data were submitted to descriptive and inferential analyses (bivariate and multivariate). Separate ANOVA was used to calculate the differences in the BRIEF-P scales and clinical indices between the two groups according to sample type (normotypical development sample vs. clinical sample) and informant. The analyses were conducted using the SPSS software, version 29.0.2.0.

## 3. Results

The descriptive data (see Table 5) show that the clinical population exhibits greater difficulties across all dimensions assessed by the BRIEF-P, both in the home and school environments. The largest discrepancies are observed in indices that integrate multiple executive domains, such as the Emergent Metacognition Index and the Global Executive Composite, reflecting more complex and widespread impairments in self-regulation and everyday executive functioning.

**Figure 1:**
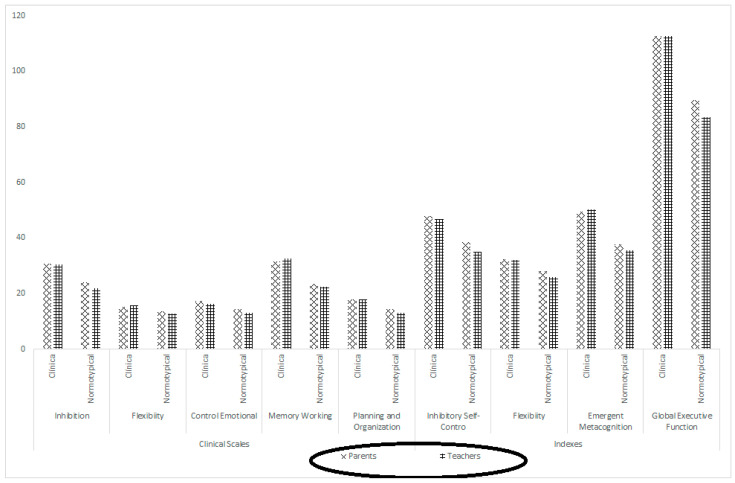
Mean BRIEF-P scores obtained by the two subsamples (clinical vs. neurotypical) according to the different informants (parents vs. teachers). Source: BRIEF-P (Spanish adaptation). Sample: (i) clinical, (a) parents = 107 and (b) teachers = 98; and (ii) normotypical, (a) parents = 1077 and (b) teachers = 902.

Figure 1 shows the mean BRIEF-P scores obtained by the two subsamples (clinical vs. neurotypical) according to the different informants (parents vs. teachers).

The analysis of variance (ANOVA) (see Table 6) robustly confirms the differences previously observed in the descriptive data. Statistically significant differences (*p* < 0.001) were found between the clinical and normotypical populations across all BRIEF-P clinical scales and indices, as reported by both parents and teachers.

The highest F-values were observed in the following:

(i) Working memory: (a) parents, [F = 195.76, *p* < 0.001]; (b) teachers, [F = 199.63, *p* < 0.001].
(ii) Emergent Metacognition Index: (a) parents, [F = 176.15, *p* < 0.001]; (b) teachers, [F = 187.87, *p* < 0.001].
(iii) Executive Function Global: (a) parents, [F = 168.07, *p* < 0.001]; (b) teachers, [F = 207.47, *p* < 0.001].

These three variables reflect core components of executive functioning and are particularly sensitive to detecting difficulties in the clinical population (Miyake et al., 2000).

Similarly, variables such as inhibition, planning, and organization, and composite indices like the Inhibitory Self-Control Index also show highly significant differences (F > 100 in all cases), indicating consistent impairments in self-regulation and planning.

All differences were statistically significant (*p* < 0.001), confirming a disrupted executive functioning profile in the clinical population that is consistent across both evaluation contexts (home and school).

The results reflect small-to-moderate effect sizes (see Table 7), depending on the clinical scale and indices analyzed. In general, we observed the following:

(i) Teachers tend to report larger effect sizes than parents, especially in global executive functions and metacognition.
(ii) The highest estimates are observed in working memory, emergent metacognition, and global executive function, suggesting that these dimensions better capture the differences between groups or conditions analyzed.
(iii) Scales with lower effect sizes (such as flexibility) may indicate that group differences are less pronounced in that domain.
(iv) The most affected executive functions (by group, intervention, or condition) are working memory, emergent metacognition, and global executive functioning.
(v) Teachers tend to detect stronger effects than parents in most dimensions, which may reflect greater observational sensitivity in structured school contexts.
(vi) Although some scales, such as flexibility or emotional control, show lower effect sizes, they are not null, which still adds value to their analysis.

### 3.1. Parent Evaluation (Clinical Sample vs. Neurotypical Sample)

On all BRIEF-P scales, participants in the clinical sample obtained higher mean scores than those in the neurotypical group, indicating a greater level of executive function problems/difficulties. The differences are statistically significant in all cases (*p* < 0.001), with effect sizes ranging from small to large depending on the scale.

The areas with the greatest impact are (i) working memory, [F = 195.76; η^2^ = 0.142], with a mean difference of 8.16 points in favor of the clinical group; (ii) Emerging Metacognition Index, [F = 176.15; η^2^ = 0.13], with a mean difference of 11.75 points; and (iii) Global Executive Composite, [F = 168.07; η^2^ = 0.124], with a mean difference of 23.

The areas with moderate impact are (i) inhibition scale, [F = 131.49; η^2^ = 0.10], with a mean difference of 6.82; (ii) planning and organization, [F = 106.07; η^2^ = 0.082], with a mean difference of 3.58; and (iii) Inhibitory Self-Control Index, [F = 116.75; η^2^ = 0.09], with a mean difference of 9.57.

The areas with lesser but still significant impact are (i) emotional control, [F = 54.72; η^2^ = 0.044], with a mean difference of 2.75; (ii) flexibility (clinical scale), [F = 25.19; η^2^ = 0.021], with a mean difference of 1.67 points; and (iii) Flexibility Index, [F = 55.18; η^2^ = 0.045], with a mean difference of 4.42.

### 3.2. Teachers Evaluation (Clinical Sample vs. Neurotypical Sample)

Both parents and teachers detect statistically significant differences between participants in the clinical group and the neurotypical group across all BRIEF-P scales, but the magnitude of the differences tends to be slightly greater according to teachers in most domains.

The areas with the greatest impact are (i) working memory, [F = 199.632; η^2^ = 0.167], with a mean difference of 9.98; (ii) Emerging Metacognition Index, [F = 187.869; η^2^ = 0.158], with a mean difference of 14.76; and (iii) Global Executive Composite, [F = 207.474; η^2^ = 0.172], with a mean difference of 29.46.

The areas with moderate impact are (i) inhibition, [F = 155.526; η^2^ = 0.135], with a mean difference of 9.38; (ii) planning and organization, [F = 140.485; η^2^ = 0.123], with a mean difference of 4.75; and (iii) Inhibitory Self-Control Index, [F = 134.5; η^2^ = 0.119], with a mean difference of 11.77.

The areas with lesser impact are (i) emotional control, [F = 58.764; η^2^ = 0.056], with a mean difference of 2.89; (ii) flexibility, [F = 66.499; η^2^ = 0.062], with a mean difference of 2.96; and (iii) Flexibility Index: [F = 82.615; η^2^ = 0.076], with a mean difference of 6.16.

## 4. Discussion

The objectives of the study were to analyze and compare executive functioning profiles in children with various neurodevelopmental disorders, based on reports provided by parents and teachers. This approach is grounded in the work of Sadozai et al. (2024), who, from transdiagnostic models of neurodevelopment, emphasize the importance of understanding delays in executive function from a developmental perspective. Such understanding is essential for designing early and personalized interventions that address the needs of all children with neurodevelopmental conditions, regardless of their specific diagnosis. Two main hypotheses were proposed: (i) that children with neurodevelopmental disorders exhibit executive function deficits, as measured by the BRIEF-P, in comparison to typically developing peers; and (ii) that perceptions of executive functioning differ depending on the informant, that is, between parents and teachers.

Executive functions (EFs) are key skills for cognitive and emotional self-regulation. Difficulties in EF act as a transdiagnostic marker of atypical development, present across multiple disorders and not limited to any single one (Zelazo, 2020). Mareva et al. (2024) found that executive function profiles were not directly related to specific diagnoses or associated dimensions.

Delays and/or dysfunctions in executive function (EF) have been widely documented in children with neurodevelopmental conditions (Bausela Herreras et al., 2023; Bausela Herreras, 2024b; Cordova et al., 2020; Demetriou et al., 2018; Johnson, 2012; Ozonoff & Jensen, 1999; Pardo-Salamanca et al., 2024). Otterman et al. (2019) propose that executive function difficulties occur gradually across the spectrum of ASD and ADHD, and they may precede the emergence of traits of both disorders from early childhood.

Recent studies suggest that this delay may be considered a transdiagnostic feature of these conditions. Sadozai et al. (2024) conducted a systematic review and meta-analysis including 180 studies using standardized measures such as the BRIEF-P. The results indicated a moderate effect size of EF delay across all neurodevelopmental conditions compared to controls, an effect that increased in the presence of comorbidities, DSM-5 criteria, and informant-reported measures. Comparisons between different clinical conditions showed few differences; although executive profiles can be derived, the study results appear contradictory (Antshel & Russo, 2019) (see Mirabella, 2021).

For instance, two studies (Ceruti et al., 2024; Townes et al., 2023) reported no significant differences in EF profiles between ASD and ADHD. Both groups showed lower performance than typically developing children in attention, cognitive flexibility, visuospatial skills, working memory, processing speed, and response inhibition. However, no differences were found in planning. Assessing EF in the early stages of development is complex (Spiegel et al., 2017; Townes et al., 2023). The absence of adequate assessment could hinder the early implementation of interventions aimed at improving EF (Cuartas et al., 2022; Duncan, 2023; Krivitzky et al., 2016; O’Reilly et al., 2025; Rosas et al., 2019), highlighting the importance of developing and using developmentally sensitive methods to identify transdiagnostic delays from an early age.

These findings are consistent with other disorders that support the existence of common neurobiological substrates for executive deficits. Thus, the studies by Wei et al. (2023) reported in “Editorial: Transdiagnostic correlates of executive functions in psychiatric disorders” by Heilbronner and Albrecht (2023) reinforce the importance of a transdiagnostic approach in the understanding and treatment of major psychiatric disorders (Demetriou et al., 2021; Roye et al., 2022; Sonuga-Barke et al., 2016). Feijs et al. (2024) suggest that the relationships between executive functions and psychopathology are complex and potentially non-linear, highlighting the importance of integrating EF into dimensional models and considering them as a possible transdiagnostic factor. Vaidya et al. (2020) identified three transdiagnostic executive function subtypes in children with and without ADHD and ASD, characterized by weaknesses in flexibility/emotion, inhibition, and working memory/organization. These subtypes, also present in typically developing children, better predict frontoparietal activation than DSM diagnoses and support the personalization of interventions for executive dysfunction.

This functional approach allows us to understand that, beyond traditional categorical diagnoses (such as ADHD, ASD, or dyslexia), many individuals with neurodevelopmental disorders share impairments in common domains (Michelini et al., 2024; DeYoung et al., 2024). One of the most relevant is executive functioning, whose disruption can give rise to what is known as the dysexecutive syndrome (Godefroy et al., 2010; Jones, 2020; Rabinovici et al., 2015). These functions, which are essential for adaptation to academic, social, and professional environments, may be affected to varying degrees and across different dimensions depending on the disorder, without being limited to a specific diagnostic label (Ceravolo et al., 2012; Liebermann et al., 2013; Roussel et al., 2017; Ribas et al., 2023). Understanding dysexecutive syndrome (Jones & Graff-Radford, 2021) as a transdiagnostic phenomenon allows interventions to be focused on the individual’s actual functional needs (Dalgleish et al., 2020; Dickson et al., 2023), with particular emphasis on processes such as metacognition and self-regulation (Cristofori et al., 2019; S.-F. F. Chen et al., 2016; Daunhauer et al., 2014; Hennecke & Bürgler, 2023; R. R. Lee et al., 2023; Roebers, 2017).

In relation to the informant, we can state, in general terms, that parents and teachers are reliable sources for assessing development of EFs in early childhood education. However, in agreement with other authors, there are differences and similarities in their perception of the development of EFs (Bausela Herreras, 2024a). These results may indicate that, when the teachers are the informants, they are more sensitive to development compared to the parents (Lunkenheimer et al., 2019; Ortiz Luna & Acle Tomasini, 2006; Scott et al., 2018). This study confirms that, depending on the informants, there may be differences in the assessment of executive functioning in preschool-aged children with various neurodevelopmental disorders (Albuquerque et al., 2025; Ratto et al., 2020; Schneider et al., 2020; Tamm & Peugh, 2019). Olsson et al. (2020)—in their study on patients with aphasia—point out that informant-based assessments of executive functions do not substitute for standardized neuropsychological tests; therefore, information provided by non-professional informants should always be interpreted with caution. De Los Reyes and Kazdin (2005) highlight that discrepancies between informants affect the assessment and treatment of child psychopathology. They propose the Attribution Bias Context Model as a theoretical framework to guide research and suggest exploring comparisons between different informant pairs, as well as strategies to reduce these discrepancies in clinical settings.

This study provides a clear framework for identifying dysexecutive syndrome. Since the patterns of impairment differ across conditions clinics, a structured assessment of these disorders based on established diagnostic criteria is essential (Godefroy et al., 2010).

We believe that the BRIEF-P (Gioia et al., 2016) can serve as a valuable tool for identifying executive function deficits across various neurodevelopmental disorders, based on observations from both parents and teachers. While the study’s design does not permit us to confirm the level of agreement between these informants, both tend to highlight difficulties in flexibility and emotional regulation—executive domains that appear to be affected to differing extents depending on the informant.

### 4.1. Limitations and Future Directions

This study makes a contribution to the field of executive functioning in children with neurodevelopmental disorders; however, as part of an evolving and developing line of research, it presents some areas that will be enriched in future work.

The use of unmatched samples may affect the validity of comparisons between informants, and that the ex post facto design imposes restrictions on causal inference. Inter-rater agreement is addressed on page 59 of the Spanish BRIEF-P (Gioia et al., 2016). The results obtained are consistent with those reported by other researchers (Albuquerque et al., 2025; Andersen et al., 2024; Ratto et al., 2020; Schneider et al., 2020). Reducing all childhood difficulties to a single diagnosis carries the risk of overdiagnosis and inadequate interventions. Therefore, assessment should be comprehensive, taking into account overall development, the family and school context, and the possibility of coexisting conditions. Recognizing the diversity of neurodevelopmental disorders and conducting a careful differential diagnosis are key to guiding appropriate support.

The size of some clinical groups, although sufficient to detect significant differences at a global level, opens the possibility of expanding these samples in subsequent research to further explore specific executive profiles according to diagnosis. A pilot study has been conducted and published on two of the subsamples—ASD and ADHD (Bausela Herreras, 2020).

The difference in group sizes could potentially affect statistical power and increase the risk of Type I/II errors. To address this limitation, it was verified that the assumptions for ANOVA, such as homogeneity of variances and normality of the data, were met despite the group size imbalance, minimizing its impact on the results. To complement the ANOVA analysis and quantify the magnitude of the observed effects, the following effect size measures were calculated: Eta squared (η^2^), Epsilon squared (ε^2^), and random-effect omega squared (ω^2^). Likewise, the use of informant questionnaires, gathering perspectives from parents and teachers, provides a valuable ecological view of executive behavior in everyday contexts, which can be complemented in future studies with direct behavioral assessments of the participants themselves (self-assessment) to strengthen methodological integration. Inter-rater agreement is addressed on pages 59–60 of the BRIEF-P (Gioia et al., 2016). The results obtained are consistent with those reported by other researchers (Albuquerque et al., 2025; Anderson, 2002; De Los Reyes & Kazdin, 2005; Tamm & Peugh, 2019; Toplak et al., 2013).

For future research, complementary objective measures of the child’s own executive performance could be employed. The incorporation of such complementary objective measures could help strengthen validity, although questions may still arise regarding construct validity, as previously noted by (Toplak et al., 2013).

By using a cross-sectional design, it is not possible to control for potential cohort or contextual effects. These characteristics reflect the progressive and multidimensional nature of this line of research, which currently involves doctoral students and collaborators to broaden and diversify the methodology and samples. We will further explore the following lines of work (Wang & Cheng, 2020): (i) inclusion of longitudinal data to assess the development of executive functions, (ii) a triangulation process combining informant-based and direct assessments, (iii) analysis of subgroups within the population with neurodevelopmental disorders, and (iv) cross-cultural comparisons. These additions aim to broaden the scope and robustness of the results obtained.

Identifying EF in the first years of life poses particular challenges. Escobar-Ruiz et al. (2024) reviewed advances and challenges in assessing these skills before 36 months of age, focusing on working memory, inhibition, and cognitive flexibility. Their study points to methodological limitations related to task validity at such early ages, the influence of language development, and interindividual variability, highlighting the need for adapted tools and longitudinal studies to understand the early trajectory of EF (Anderson, 2002; Blair, 2016).

### 4.2. Contribution to the Field

In this way, the current study constitutes a foundation that allows for the identification of general patterns and paves the way for future research with greater depth and scope.

In summary, this study may represent an opportunity to continue advancing and enriching knowledge about executive function profiles during the early stages of the life cycle, when executive functions are most interconnected (see the model by (Miyake et al., 2000)), reaffirming the importance of this work in the transdiagnostic study of neurodevelopmental disorders in early life (Brignell et al., 2022; McCraw et al., 2024; Michelini et al., 2024; Peng et al., 2025; Pezzoli et al., 2023; Williams et al., 2024).

This work supports the conception of dysexecutive syndrome as a transdiagnostic feature and reinforces the need to implement early screening procedures for executive functions. Additionally, it provides validation data for the BRIEF-P in Spanish (Gioia et al., 2016; Bausela Herreras & Luque Cuenca, 2017), thus contributing to the availability of tools adapted to different linguistic contexts and facilitating the development of cross-cultural research, thereby improving the comparability of results at the international level (Duku & Vaillancourt, 2014; Rai et al., 2017; San Diego et al., 2023).

Various interventions have shown efficacy in promoting EF development in neurodevelopmental disorders populations (Etokabeka, 2024). A few studies (Gavrilova et al., 2024; O’Reilly et al., 2025; Kubota et al., 2023; Liu et al., 2025) demonstrated that both the duration of preschool attendance and the quality of teacher–child interactions significantly influence EF development, underscoring the role of educational context and high-quality pedagogical practices in enhancing these skills.

Overall, these findings support transdiagnostic models of neurodevelopment and highlight the importance of early assessment of executive function to guide interventions that optimize the development of children with neurodevelopmental disorders. This study is in line with current research that supports the transdiagnostic approach, such as the study carried out by researchers from the Biomedical Research Networking Center in Mental Health (CIBERSAM), in collaboration with the Mood and Anxiety Disorders Imaging Group at IDIBAPS, who have led the creation of the first transdiagnostic brain atlas that identifies specific gray matter patterns associated with major mental disorders (Fortea et al., 2025; Salagre et al., 2019).

## Figures and Tables

**Table 1:** Distribution of participants according to the type of sample and informant (own elaboration).

Sample	Informant		
	Parents	Teachers	Subtotal
Clinical	107	98	205
Normotypical	1077	902	1979
Subtotal	1184	1000	2184

Source: BRIEF-P (Spanish adaptation).

**Table 2:** Distribution of participants according to age group, informant, and sample (own elaboration).

Informant	Sample	Age (Years)				
		2	3	4	5	Subtotal
Parents	Clinical	10	26	32	39	107
	Normotypical	156	262	339	320	1077
	Subtotal	166	288	371	359	1184
Teachers	Clinical	9	24	32	33	98
	Normotypical	118	208	295	281	902
	Subtotal	127	232	327	314	1000

Source: BRIEF-P (Spanish adaptation).

**Table 3:** Distribution of participants according to sex, informant, and sample (own elaboration).

Informant	Sample	Sex		
		Man	Woman	Subtotal
Parents	Clinical	82	25	107
	Normotypical	567	510	1077
	Subtotal	649	535	1184
Teachers	Clinical	74	24	98
	Normotypical	475	427	902
	Subtotal	549	451	1000

Source: BRIEF-P (Spanish adaptation).

**Table 4:** BRIEF-P adapted from (Gioia et al., 2016): clinical scales and indices (description and examples) (own elaboration).

Clinical Scales	Description	Examples
Inhibition	Assesses problems with controlling impulses and behavior, and difficulties stopping or appropriately regulating actions in specific moments or contexts.	During activities, the child is easily distracted from their goal.
Flexibility	Assesses difficulties in voluntarily shifting between situations or activities and solving problems in a flexible manner.	Has trouble switching from one activity to another.
Emotional control	Evaluates problems with adequately regulating or modulating emotional responses according to situational demands.	Becomes upset very easily.
Working memory	Assesses difficulties in holding information in mind to complete a task or provide an appropriate response.	Has trouble remembering things even after a short period of time.
Planning and organization	Assesses problems with anticipating future events or consequences.	Has trouble finding their belongings in their room or play area, even when given specific directions.
Indices	Description	
Inhibitory Self-Control Index	Sum of raw scores from the inhibition and emotional control scales.	
Flexibility Index	Sum of raw scores from the flexibility and emotional control scales.	
Emergent Metacognition Index	Sum of raw scores from the working memory and planning and organization scales.	

Source: BRIEF-P (Spanish adaptation).

**Table 5:** Descriptive statistics of participants on BRIEF-P clinical scales and indices by informant and sample type (own elaboration).

Clinical Scales and Indices	Sample	Informant					
		Parents			Teachers		
		Mean	Standard Deviation	Standard Error	Mean	Standard Deviation	Standard Error
Inhibition	Clinical	30.74	7.25	0.70	30.48	8.21	0.83
	Normotypical	23.92	5.71	0.17	21.91	6.25	0.21
	Subtotal	24.54	6.18	0.18	22.75	6.95	0.22
Flexibility	Clinical	15.15	4.08	0.39	15.66	5.24	0.53
	Normotypical	13.48	3.20	0.10	12.7	3.16	0.11
	Subtotal	13.63	3.32	0.10	12.99	3.53	0.11
Emotional control	Clinical	17.23	4.21	0.41	16.33	5.31	0.54
	Normotypical	14.48	3.62	0.11	13.14	3.73	0.12
	Subtotal	14.73	3.76	0.11	13.45	4.03	0.13
Working memory	Clinical	31.51	7.70	0.75	32.45	8.92	0.90
	Normotypical	23.35	5.53	0.17	22.47	6.35	0.21
	Subtotal	24.09	6.21	0.18	23.45	7.27	0.23
Planning and organization	Clinical	17.96	4.36	0.42	17.89	5.47	0.55
	Normotypical	14.38	3.33	0.10	13.14	3.53	0.12
	Subtotal	14.71	3.58	0.10	13.61	4.02	0.13
Inhibitory Self-Control Index	Clinical	47.97	10.26	0.99	46.81	12.35	1.25
	Normotypical	38.4	8.57	0.26	35.04	9.181	0.31
	Subtotal	39.27	9.16	0.27	36.2	10.15	0.32
Flexibility Index	Clinical	32.38	6.96	0.67	31.99	9.55	0.97
	Normotypical	27.96	5.76	0.18	25.83	5.93	0.20
	Subtotal	28.36	6.01	0.18	26.44	6.63	0.21
Emergent Metacognition Index	Clinical	49.48	11.51	1.11	50.34	13.92	1.41
	Normotypical	37.73	8.41	0.26	35.61	9.60	0.32
	Subtotal	38.79	9.36	0.27	37.06	11.00	0.35
Executive Function Global	Clinical	112.6	21.61	2.09	112.81	25.88	2.61
	Normotypical	89.61	17.03	0.52	83.35	18.37	0.61
	Subtotal	91.69	18.69	0.54	86.24	21.12	0.67

Source: BRIEF-P (Spanish adaptation). Sample: (i) clinical, (a) parents = 107 and (b) teachers = 98; and (ii) normotypical, (a) parents = 1077 and (b) teachers = 902.

**Table 6:** ANOVA of participants on BRIEF-P clinical scales and indices by informant and sample type (own elaboration).

ANOVA	Parents		Teachers	
	F	Sig.	F	Sig.
Inhibition	131.486	<0.001 ***	155.526	<0.001 ***
Flexibility	25.189	<0.001 ***	66.499	<0.001 ***
Emotional control	54.718	<0.001 ***	58.764	<0.001 ***
Working memory	195.763	<0.001 ***	199.632	<0.001 ***
Planning and organization	106.074	<0.001 ***	140.485	<0.001 ***
Inhibitory Self-Control Index	116.747	<0.001 ***	134.53	<0.001 ***
Flexibility Index	55.178	<0.001 ***	82.615	<0.001 ***
Emergent Metacognition Index	176.146	<0.001 ***	187.869	<0.001 ***
Executive Function Global	168.072	<0.001 ***	207.474	<0.001 ***

Source: BRIEF-P (Spanish adaptation). Sample: (i) clinical, (a) parents = 107 and (b) teachers = 98; (ii) normotypical: (a) parents = 1077 and (b) teachers = 902. *** *p* < 0.001 (highly significant).

**Table 7:** Effect sizes from ANOVA on BRIEF-P clinical scales and indices by informant and sample type (own elaboration).

Clinical Scales and Indices	Effect Sizes from ANOVA	Parents			Teachers		
		Point Estimate	95% Confidence Interval		Point Estimate	95% Confidence Interval	
			Lower	Upper		Lower	Upper
Inhibition	Eta squared	0.1	0.07	0.133	0.135	0.098	0.174
	Epsilon squared	0.099	0.069	0.132	0.134	0.097	0.173
	Fixed-effect omega squared	0.099	0.069	0.132	0.134	0.097	0.173
	Random-effect omega squared	0.099	0.069	0.132	0.134	0.097	0.173
Flexibility	Eta squared	0.021	0.008	0.04	0.062	0.037	0.093
	Epsilon squared	0.02	0.007	0.039	0.062	0.036	0.092
	Fixed-effect omega squared	0.02	0.007	0.039	0.061	0.036	0.092
	Random-effect omega squared	0.02	0.007	0.039	0.061	0.036	0.092
Emotional control	Eta squared	0.044	0.024	0.069	0.056	0.031	0.085
	Epsilon squared	0.043	0.023	0.068	0.055	0.03	0.084
	Fixed-effect omega squared	0.043	0.023	0.068	0.055	0.03	0.084
	Random-effect omega squared	0.043	0.023	0.068	0.055	0.03	0.084
Working memory	Eta squared	0.142	0.108	0.178	0.167	0.127	0.208
	Epsilon squared	0.141	0.107	0.178	0.166	0.126	0.207
	Fixed-effect omega squared	0.141	0.107	0.177	0.166	0.126	0.207
	Random-effect omega squared	0.141	0.107	0.177	0.166	0.126	0.207
Planning and organization	Eta squared	0.082	0.055	0.113	0.123	0.088	0.161
	Epsilon squared	0.082	0.054	0.113	0.123	0.087	0.161
	Fixed-effect omega squared	0.082	0.054	0.112	0.122	0.087	0.161
	Random-effect omega squared	0.082	0.054	0.112	0.122	0.087	0.161
Inhibitory Self-Control Index	Eta squared	0.09	0.061	0.122	0.119	0.084	0.156
	Epsilon squared	0.089	0.061	0.121	0.118	0.083	0.156
	Fixed-effect omega squared	0.089	0.06	0.121	0.118	0.083	0.156
Flexibility Index	Eta squared	0.045	0.024	0.07	0.076	0.048	0.109
	Epsilon squared	0.044	0.024	0.069	0.076	0.047	0.109
	Fixed-effect omega squared	0.044	0.024	0.069	0.075	0.047	0.108
	Random-effect omega squared	0.044	0.024	0.069	0.075	0.047	0.108
Emergent Metacognition Index	Eta squared	0.13	0.096	0.165	0.158	0.12	0.199
	Epsilon squared	0.129	0.096	0.164	0.158	0.119	0.198
	Fixed-effect omega squared	0.129	0.096	0.164	0.157	0.119	0.198
	Random-effect omega squared	0.129	0.096	0.164	0.157	0.119	0.198
Executive Function Global	Eta squared	0.124	0.092	0.16	0.172	0.132	0.213
	Epsilon squared	0.124	0.091	0.159	0.171	0.131	0.212
	Fixed-effect omega squared	0.124	0.091	0.159	0.171	0.131	0.212
	Random-effect omega squared	0.124	0.091	0.159	0.171	0.131	0.212

Source: BRIEF-P (Spanish adaptation). Sample: (i) clinical, (a) parents = 107 and (b) teachers = 98; and (ii) normotypical, (a) parents = 1077 and (b) teachers = 902.

## Data Availability

These data have been provided to complement the information presented in the text, while fully respecting the confidentiality and privacy established as co-authors of the BRIEF-P Spain adaptation, as well as the commitments signed with TEA Ediciones Publishing. These data were managed with due ethical consideration and in accordance with the established agreements, ensuring the protection of sensitive information.

## Abbreviations

The following abbreviations are used in this manuscript:

ADHD	Attention-deficit/hyperactivity disorder
ASD	Autism spectrum disorder
BRIEF-P	Behavior Rating Inventory of Executive Function in Preschool
EFs	Executive functions
